# Social anxiety is associated with heart rate but not gaze behavior in a real social interaction

**DOI:** 10.1016/j.jbtep.2020.101600

**Published:** 2021-03

**Authors:** Lara Rösler, Stefan Göhring, Michael Strunz, Matthias Gamer

**Affiliations:** Department of Psychology, Julius Maximilians University of Würzburg, Würzburg, Germany

**Keywords:** Social anxiety, Eye gaze, Gaze perception, Heart rate, Skin conductance level, Heart rate variability

## Abstract

**Background and objectives:**

Much of our current understanding of social anxiety rests on the use of highly restricted laboratory experiments. Latest technological developments now allow the investigation of eye movements and physiological measures during real social interactions. Considering the wealth of conflicting findings on gaze behavior in social anxiety, the current study aimed at elucidating the modulation of gaze patterns in a naturalistic setting.

**Methods:**

We introduced 71 participants with differing social anxiety symptoms to a waiting room situation while recording heart rate, electrodermal activity and eye movements using mobile technology.

**Results:**

We observed fewer fixations on the head of the confederate in the initial waiting phase of the experiment. These head fixations increased when the confederate was involved in a phone call and subsequently initiated an actual conversation. Contrary to gaze-avoidance models of social anxiety, we did not observe any correlations between social anxiety and visual attention but an elevated heart rate in participants with high social anxiety.

**Limitations:**

Although social anxiety varied considerably in the current sample and reached clinically relevant levels in one third of participants, formal clinical diagnoses were not available.

**Conclusions:**

The current findings suggest that gaze avoidance might only occur in specific situations or very high levels of social anxiety. Fear of eye contact could at times represent a subjectively experienced rather than an objectively measurable feature of the disorder. The observation of elevated heart rate throughout the entire experiment indicates that physiological hyperactivity might constitute a cardinal feature of social anxiety.

## Highlights

• Socially anxious individuals do not show altered gaze during a social interaction.
• Social anxiety is associated with elevated heart rate in this naturalistic setting.
• Gaze reductions might be restricted to particularly threatening situations.
• Physiological hyperactivity could constitute a more general feature of the disorder.

## 1 Introduction

Understanding social contexts is essential for human interactions. At the center of interpreting the social dynamics of a scene lies an attentional bias towards the fellow humans taking part in it (Birmingham & Kingstone, 2009). Various laboratory studies have shown increased gaze towards human bodies and, in particular, faces, independent of the task at hand (Flechsenhar & Gamer, 2017) or the surrounding visual information (Birmingham, Bischof, & Kingstone, 2009; Boggia & Ristic, 2015; End & Gamer, 2017; Rösler, End, & Gamer, 2017). However, recent advances in technology have enabled researchers to move the studies on social attention from laboratory settings to the real world. After all, the viewing of stimuli on a PC screen lacks many aspects a genuine social encounter entails. A major concern of the proponents of ecological validity (Kingstone, 2009; Kingstone, Smilek, & Eastwood, 2008; Risko, Laidlaw, Freeth, Foulsham, & Kingstone, 2012; Smilek, Birmingham, Cameron, Bischof, & Kingstone, 2006) is that most of the psychological stimulation material does not adequately approximate the complexity of daily life situations. While participants are known to preferentially fixate eyes of schematic or photographed faces presented in isolation (e.g. Friesen & Kingstone, 1998; Frischen, Bayliss, & Tipper, 2007; Langton, Watt, & Bruce, 2000), more intricate visual elements of real world scenes might draw attention away from the eyes. Indeed, several studies have shown substantially reduced overt attention to humans when participants are moved from the lab into environments with higher ecological validity (Foulsham, Walker, & Kingstone, 2011; Hayward, Voorhies, Morris, Capozzi, & Ristic, 2017; Laidlaw, Foulsham, Kuhn, & Kingstone, 2011). Accordingly, participants look significantly less at an actual person sitting in a waiting room with them than if that same person is not physically present but displayed on a computer screen. These gaze reductions possibly stem from an active avoidance of a social interaction (Laidlaw et al., 2011). Similarly, when asked to freely walk around the campus, people displayed significantly less overt attention to humans than when asked to view videos of these campus walks in the laboratory (Foulsham et al., 2011). These results argue for further investigations of gaze behavior in everyday situations to clarify which mechanisms might underlie these observed reductions.

While an attentional shift towards humans is vital for various higher socio-cognitive tasks, several psychiatric disorders display altered processing of human features. Social anxiety, which is characterized by a penetrating fear of social encounters and social-evaluative situations (Clark & Wells, 1995) and typically has a life-long impact on the relationships of the affected individuals (Wittchen, Stein, & Kessler, 1999), is assumed to feature differential processing of social information as a core symptom (Wells et al., 1995). There is, however, vast disagreement regarding the characteristics of these gaze behavior alterations (Chen & Clarke, 2017). Studies using emotional faces as probes to investigate attentional biases have shown that socially anxious patients predominantly avoid threatening stimuli (Chen et al., 2002, 2020; Mansell, Clark, Ehlers, & Chen, 1999; Weeks, Howell, Srivastav, & Goldin, 2019), while heightened processing of emotional stimuli in social anxiety has been reported elsewhere (Asmundson & Stein, 1994; Chen, Yao, Qian, & Lin, 2016; Gilboa-Schechtman, Foa, & Amir, 1999; Kret, Stekelenburg, de Gelder, & Roelofs, 2017). In addition to these alterations on the behavioral level, measures of electrodermal activity can shed light on the activation of the sympathetic nervous system in social anxiety. In socially anxious groups, elevated skin conductance levels have been observed while listening to threatening scenarios (Lang & McTeague, 2009), giving a speech in front of an audience (Deiters, Stevens, Hermann, & Gerlach, 2013) and watching anxiety-provoking situations (Panayiotou, Karekla, Georgiou, Constantinou, & Paraskeva-Siamata, 2017). However, a potential association between electrodermal activity and social anxiety has not been consistently reported and seems to be contingent on the situational context (see Panayiotou et al., 2017; Puigcerver, Martinez-Selva, Garcia-Sanchez, & Gomez-Amor, 1989; White & Graham, 2018).

Up to date, only a handful of studies investigated gaze behavior in social anxiety during a seemingly real interaction and, again, provided conflicting results (Farabee, Holcom, Ramsey, & Cole, 1993; Howell, Zibulsky, Srivastav, & Weeks, 2016; Langer, Lim, Fernandez, & Rodebaugh, 2017). Of these, two studies used video recordings to infer gaze patterns (Farabee et al., 1993; Langer et al., 2017) and the only study which actually recorded eye movements tested a webcam interaction design in only 20 participants (Howell et al., 2016). Considering that viewing preferences of social features change from lab to real-world environments (Foulsham et al., 2011; Hayward et al., 2017; Laidlaw et al., 2011; Rubo, Huestegge, & Gamer, 2019), it seems crucial to assess gaze behavior in socially anxious participants with adequate methodology during an actual social interaction. Since elevated skin conductance levels in social anxiety are primarily associated with real eye contact rather than the observation of photographed faces (Myllyneva, Ranta, & Hietanen, 2015), an investigation of gaze and physiological responses in social anxiety towards different degrees of social interaction in a real-world setting will help to clarify the mechanisms underlying anxious behavior. The aim of the present studies was hence to investigate whether the presence or absence of a potential social interaction influences gaze allocation and whether social anxiety modulates viewing behavior and physiological responses across these different social situations. We therefore measured eye gaze, heart rate and electrodermal activity (EDA) of participants with different degrees of (sub-)clinical social anxiety using a mobile eye-tracking device and mobile physiological sensors. Throughout the entire recording, participants were seated in a room with a male confederate whom they believed to be another participant. During this time, they were not aware that the experiment had already started. To modulate the degree to which a social interaction was possible, the confederate received a phone call 2 min into the experiment, rendering him unavailable for a conversation. He subsequently addressed the participant directly and initiated a semi-stereotyped conversation. Based on the results of the waiting room study of Laidlaw et al. (2011), we expected that head fixations would be most reduced in the initial waiting phase when an interaction had to be actively avoided. Since an interaction is impossible during the phone phase and thus does not have to be actively avoided, we expect head fixations to then increase and be highest during the interaction where mutual gaze is normative. Additionally, we expected that higher levels of social anxiety would be associated with reduced social gaze accompanied by increased physiological arousal throughout all conditions but most prominently during the interaction phase.

## 2 Methods

### 2.1 Participants

**Fig. 1:**
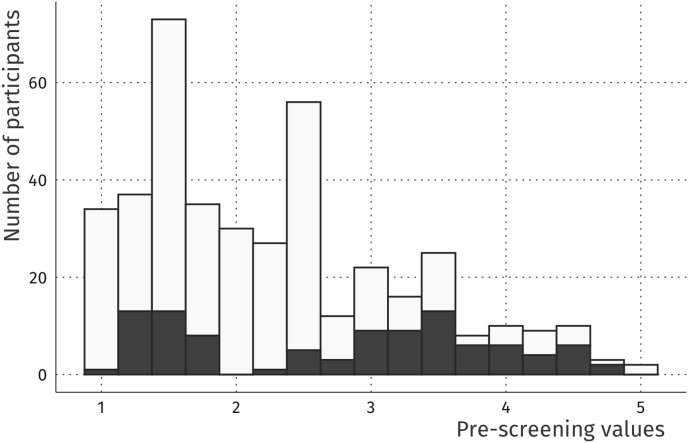
**Pre-screening values obtained during online recruitment.** Higher values indicate higher overlap with DSM-IV criteria of social anxiety. People who participated in the current study are highlighted in dark grey. Note that not everyone who participated in the pre-screening accepted an invitation to the lab.

Prior to recruitment, we asked potential participants to fill in several questionnaires via an online recruiting platform hosted by the university which predominantly but not exclusively targets students. The pre-screening enabled us to include a wide spectrum of social anxiety manifestations in our sample. When planning the study, we aimed at examining approximately twice as many participants as in previous studies focusing on the influence of individual differences on gaze patterns in naturalistic situations (e.g., *n* = 26, Laidlaw et al., 2011; *n* = 32 Experiment 1, Freeth, Foulsham, & Kingstone, 2013; *n* = 26 Real-life group, Rubo et al., 2020; *n* = 36,; Vabalas & Freeth, 2016). In order to reach an approximately uniform distribution of social anxiety traits in our sample and since we expected a substantial number of dropouts due to recording problems or suspicion with our cover story, we initially invited 98 participants from more than 400 people who participated in a previous online screening on social anxiety traits (see Fig. 1). A few participants had to be excluded because of various issues with data acquisition or quality, including too large gaze drifts evoked by moving glasses (*n* = 3), hardware recording issues on the day of testing (*n* = 5), too many missing valid fixations (>30% of recorded frames, *n* = 9) and insufficient quality of the electrocardiogram (ECG) data (*n* = 4). Additionally, unforeseeable events during the recording led to further exclusions, to be specific the non-permitted use of mobile phones (*n* = 3), the initiating of a conversation with the confederate during the waiting phase of the experiment (*n* = 2), disbelief in the cover story of the experiment (*n* = 3) and acquaintance with the confederate (*n* = 1). Some participants met two of these exclusion criteria, resulting in a sample size of 71 for the final analysis.

The main analyses are based on an ANCOVA approach including one (in case of physiological variables) or two within-subject factors (for eye-tracking data), respectively, and the mean-centered covariate SIAS score. Formal power analyses are difficult for such a design, but a universal approach for estimating the power of a general linear model using the R package pwr (Champely, 2020) indicate a power between .90 and .99 for detecting main and interactions effects of medium size (*f*^*2*^ = 0.15) with the current sample size (*n* = 71) on an a priori significance level of α = 0.05.

Since we preselected participants according to their social anxiety and higher levels are more prevalent among females than males (McLean, Asnaani, Litz, & Hofmann, 2011), 61 of the participants were female (mean age = 24.53 years, *SD* = 6.37 years). The study was approved by the local ethics committee. Each participant provided written informed consent prior to the start of the experiment for the study they believed to take part in. Participants were informed about the purpose of the actual experiment and their rights to withdraw consent after recordings were complete. All participants received monetary compensation for their participation.

As part of the experiment, participants completed the following questionnaires to allow for a comprehensive characterization of the sample regarding anxiety, autism and general personality traits (see Table 1): The German versions of the Social Interaction Anxiety Scale (SIAS; English original version: Mattick & Clarke, 1998; German version: Stangier, Heidenreich, Berardi, Golbs, & Hoyer, 1999), the Brief Fear of Negative Evaluation Scale – Revised (BFNE; English original version: Nicholas Carleton, McCreary, Norton, & G Asmundson, 2006; German version: Reichenberger et al., 2016), the short version of the Autism Spectrum Quotient (AQ; English original version: Baron-Cohen, Wheelwright, Skinner, Martin, & Clubley, 2001; German version: Freitag et al., 2007), the trait scale of the State-Trait Anxiety Inventory (STAI; English original version: Spielberger, Gorsuch, Lushene, Vagg, & Jacobs, 1983; German version: Laux, Glanzmann, Schaffner, & Spielberger, 1981) and the short version of the Big Five Inventory (BFI; English original version: John, Donahue, & Kentle, 1991; German version: Rammstedt & John, 2005). The range of SIAS scores in the current sample indicates that we were successful in recruiting participants ranging from very low social anxiety to levels also observed in clinical samples (see Table 1). In fact, 26 participants in our sample surpassed a cut-off score of 30, which has been proposed for the differentiation of patients with social anxiety disorders from healthy participants as well as other patient groups (Stangier et al., 1999).

**Table 1:** Sample questionnaire descriptives.

	Mean	SD	Minimum	Maximum^a^
SIAS	25.96	15.78	6	71/80
BFNE	38	12.23	13	60/60
STAI	42.59	12.33	24	73/80
AQ	8.87	5.30	1	22/33
BFI Extraversion	3.37	1.06	1.25	5/5
BFI Agreeableness	2.83	0.72	1	4.5/5
BFI Conscientiousness	3.77	0.65	2.25	5/5
BFI Neuroticism	3.45	0.88	1	5/5
BFI Openness	4.10	0.66	1.8	5/5

a Highest score obtained in our sample out of the maximum score possible.

### 2.2 Measurement devices

We used mobile SensoMotoric Instruments ETG 2W eye-tracking glasses to record eye movements of both eyes with a frame rate of 60 Hz. The front camera of the glasses had a resolution of 1280 x 960 pixels and recorded the field of view with a frame rate of 24 Hz. To measure heart rate and heart rate variability, we attached a Movisens mobile EcgMove 3 Sensor to the sternum of participants by means of a chest belt. The ECG raw signal was sampled with a frequency of 1024 Hz. We additionally used a Movisens mobile EdaMove 3 Sensor attached to the thenar and hypothenar eminences of the participant's non-dominant hand to record electrodermal activity with a sampling rate of 32 Hz by a constant voltage system (0.5 V). Questionnaires were filled in on a Dell Latitude 11″ Pro 5170 Tablet using the software Presentation (Neurobehavioral Systems, 2016).

### 2.3 Procedure

**Fig. 2:**
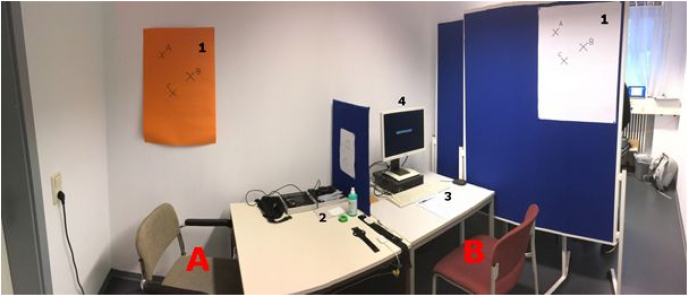
**Experimental setup.** A = Participant position, B = Confederate position, 1 = Posters used for three-point eye-tracker calibration and validation, 2 = Measurement devices, 3 = Confederate questionnaires, 4 = Experimental PC with putative experiment message.

**Fig. 3:**
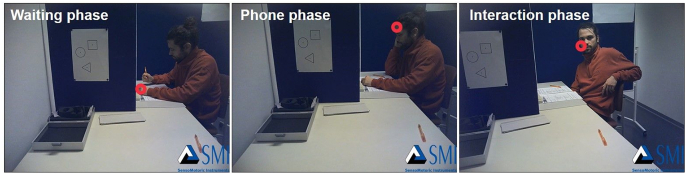
**Experimental procedure.** Exemplary images of each of the three experiment phases as viewed by the participant.

Participants expected to take part in an experiment which investigated pupil size in response to visual stimulation. Upon their arrival at the institute, the experimenter informed participants that the prior participant (confederate) experienced a slight delay and therefore still filled in some questionnaires. To allegedly save time, ECG- and EDA-electrodes as well as mobile eye-tracking glasses were attached to the participant in the meantime. The eye-tracker was subsequently calibrated by using three points of reference on a poster (see Fig. 2). Upon successful calibration, eye-tracking recording started. Simultaneously, successful calibration cued the confederate to remark that one of the questionnaires had not been copied properly. To minimize movements, as all devices were already attached and calibrated, the participant was asked to take a seat facing the confederate (see Fig. 3). Under the pretense of fetching a new questionnaire, the experimenter left the room, which marked the onset of the actual experiment. During the first 2 minutes, which will be referred to as the waiting phase throughout the article, confederate and participant did not interact but the confederate was occupied filling in the remaining questionnaires. After 2 minutes, he received a phone call, allegedly by a friend but in truth by the experimenter, which lasted approximately another 2 minutes (phone phase). Upon hanging up the phone, the confederate initiated the 2-minutes interaction phase discussing previous experiences with psychological experiments until the experimenter entered the room again (see https://osf.io/ys2uf/ for the question catalogue of the interaction phase). Although we attempted to generate experimental phases with a length of exactly 2 minutes, these durations varied slightly due to several reasons (e.g., time to establish and finish the phone call, responses of the participant in the interaction phase). On average, the waiting phase lasted 114.72 s (*SD* = 8.62 s), the phone phase 119.58 s (*SD* = 4.64 s) and the interaction phase 115.27 s (*SD* = 7.88 s) respectively.

Throughout all three phases, we measured ECG, EDA and gaze with the previously attached mobile devices. After completion of the interaction phase, the three-point poster was used to re-assess the initial calibration and enable a later drift correction. The participants were then informed about the actual purpose of the study and asked to fill in a sociodemographic (inquiring about age, gender and occupation) and the other trait questionnaires (see Table 1). All participants were asked whether they believed the cover story. In total, three participants had been suspicious throughout the experiment and were therefore excluded from the analyses.

### 2.4 Data processing

Eye-tracking and video data were handled within the BeGaze software (Version 3.6) and, as an initial step, field-of-view videos were exported as full-length AVI-files at 30 Hz excluding any fixation data. Fixation coordinates for each frame were exported separately as CSV-files. The AVI-files were then converted into single pictures per video frame using MATLAB (The MathWorks Inc, 2018). As many participants displayed a slight gaze drift tested by the final three-point validation at the end of the experiment, this drift was corrected per participant. For this purpose, the distance between the three actual fixations and points to be fixated was measured manually using the GIMP compass tool (Version 2.8; The GIMP Documentation Team, 2015). The mean of the deviations was then used for a linear gaze drift correction of the extracted fixation coordinates. If the mean deviation was larger than 200 pixels, equivalent to half of the confederate's head size, the participant was excluded entirely from the analyses. Using MATLAB, the drift-corrected coordinates were projected onto the video frames creating a ring shape with an inner radius of 9 pixels and an outer circle diameter of 25 pixels around the fixation (see Fig. 3 for an example image). We evaluated fixations manually in an application created specifically for this coding purpose at 6 Hz (i.e., every fifth frame to reduce the time spent on the still lengthy manual procedure). One independent coder who was not involved with the design or implementation of the study rated which region of interest (ROI; confederate head, body or surroundings) was fixated or if no valid fixation was present in the respective frame. A subset of participants (*n* = 6) was rated by a second rater to establish that sufficient inter-rater reliability was present (Cohen's κ = 0.93). Based on these ratings, fixation proportion per ROI for all valid fixations within a phase were calculated for each participant.

EDA and ECG raw data were initially read and exported to CSV-files via the Movisens unisens4matlab toolbox. During this step, ECG data were downsampled to 512 Hz. Heart beat detection was then performed using specifically developed scripts within the open-source statistical programming language R (R Core Team, 2018). First, ECG data were filtered using a 2 Hz high-pass filter in order to remove slow signal drifts. Subsequently, R-waves were detected from the ECG recordings using a semi-automatic method and R-R-intervals were converted to HR (in beats per minute, bpm) (for a similar approach see Rubo & Gamer, 2018). Heart rates below 50 or above 130 bpm were visually inspected for plausibility and corrected if necessary (i.e., in cases of undetected or erroneously detected R-waves). Subsequently, the data were transferred to a real time scale (Velden & Wolk, 1987) and the average heart rate (HR) per experimental phase was calculated within each participant.

Additionally, heart rate variability (HRV) in the high frequency (HF) band was calculated per phase using the R package RHRV (Rodríguez-Liñares, Vila, Méndez, & Lado, 2011) analogous to the procedure of Pittig and colleagues (Pittig, Arch, Lam, & Craske, 2013). In detail, Fourier analysis (window length = 60 s, window displacement = 5 s) was used to determine the spectral power density of HRV in the high frequency range of 0.15–0.40 Hz. These values were expressed in normative units by dividing them by the total absolute power across all frequencies between 0.003 and 0.40 Hz and multiplying the resulting fraction by 100. Since HRV is affected by the length of the recording period (Berntson et al., 1997), we calculated HF-HRV only for the first 90 s of each experimental phase. Since the interaction phase lasted only 75 s in one participant, statistical analyses of HF-HRV are based on 70 participants.

From the EDA recordings, we calculated the skin conductance level (SCL) for each experimental phase as the average of the low-pass filtered (1 Hz cutoff-frequency) skin conductance signal for each participant. These values are expressed in μS.

### 2.5 Statistical analyses

Data were analyzed using R (R Core Team, 2018). An a priori significance level of α = 0.05 was specified for all statistical tests. As our key question was to discern whether social anxiety impacts either gaze or physiological parameters, we computed four separate analyses of covariance (ANCOVAs) using the R package afex (Singmann, Bolker, Westfall, & Aust, 2015). For heart rate, heart rate variability and skin conductance levels, we included the mean-centered covariate SIAS score and the three-level factor experimental phase as well as their interaction term in the model. To model fixation proportions, we again included the mean-centered SIAS score and experimental phase as predictors but added the two-level factor ROI (head versus body), all possible two-way interactions and the triple interaction term to the model (for analyses of eye versus mouth region fixations see Supplemental Material and Supplemental Figs. S1 and S2). Post-hoc tests were performed using the R package emmeans (Lenth, Singmann, Love, Buerkner, & Herve, 2018). Generalized ɳ^2^ values (Bakeman, 2005) are reported as estimates of the effect size for linear model fixed effects. Statistical analyses scripts and data can be found on OSF (https://osf.io/ys2uf/). Additional analyses including gender as a covariate revealed a highly similar pattern of results (see supplementary material).

As an additional analysis, we computed split-half consistencies of head fixations per phase across participants since recent studies showed stable fixation patterns within individuals across trials and we were interested to see if we can replicate this finding in our real-life dataset (De Haas, Iakovidis, Schwarzkopf, & Gegenfurtner, 2019; Guy et al., 2019). To this end, we split the three experimental phases per participant into two equally long periods and computed head fixations within each half. We then calculated Pearson's correlation coefficients between halves across all participants for each phase.

## 3 Results

### 3.1 Gaze data

**Fig. 4:**
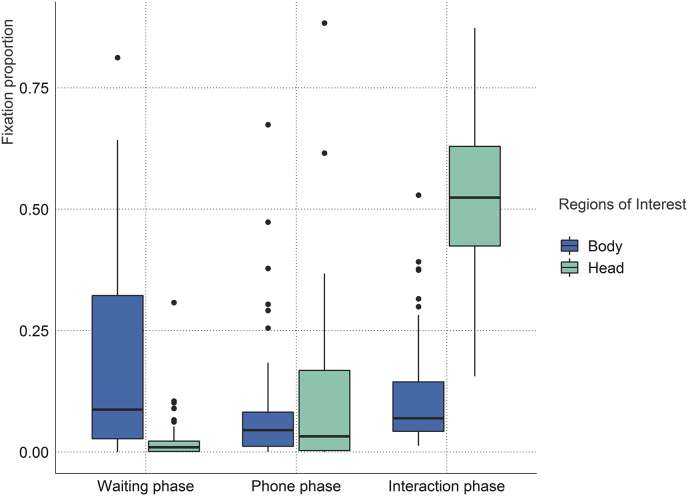
**Body and head fixation proportions during waiting, phone and interaction phase.** Outliers are denoted by black dots and defined as points further than 1.5 × interquartile range of the lower or upper hinge.

To investigate how fixation proportions on the confederate are impacted by social anxiety, experiment phase and ROI, we performed an ANCOVA with fixation proportions as the dependent variable, ROI (head versus body) and experimental phase (waiting, phone and interaction phase) as factorial predictors and SIAS scores as covariate. We found a significant main effect of ROI (*F*_(1,69)_ = 30.98, *p* < .001, η^2^ = 0.10) as there were overall considerable differences between body and head fixations (see Fig. 4 and Supplemental Table S1). A significant main effect of experiment phase (*F*_(2,138)_ = 233.87, ε = 0.95, *p* < .001, η^2^ = 0.37) is driven by higher fixation densities on the confederate during the interaction phase (see Supplemental Table S1). Importantly, we observed a significant interaction of experiment phase and ROI (*F*_(2,138)_ = 179.60, ε = 0.88, *p* < .001, η^2^ = 0.42) which mainly describes an increase of head fixations throughout the experiment (see Fig. 4 and Supplemental Table S1). Regarding potential influences of social anxiety on fixation proportion, we did not observe a significant main effect (*F*_(1,69)_ = 0.93, *p =* .339, η^2^ = 0.004) and none of the individual interactions with experimental phase and ROI, nor the triple interaction between all three predictors reached statistical significance (all *p* > .501). To test whether no effect of social anxiety on gaze behavior was more likely than social anxiety having an effect on gaze data, we compared the Bayesian ANCOVA models with and without SIAS as a covariate using the BayesFactor package (Morey, Rouder, Jamil, & Morey, 2015). Using the default prior, the resulting Bayes Factor in favor of the model without the SIAS predictor amounted to 4.26 ± 0.79% suggesting that a model which does not take social anxiety into account is approximately 4 times more likely to be the true data-generating model.

Examinations of split-half consistencies in our sample revealed strong and highly significant correlations in all phases (waiting phase: *r* = 0.56, *p* < .001; phone phase: *r* = 0.80, *p* < .001, interaction phase: *r* = 0.71, *p* < .001). Thus, viewing patterns were individually stable across participants.

### 3.2 Physiological data

**Fig. 5:**
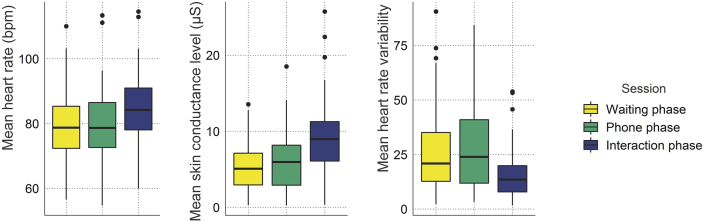
**Mean heart rate, skin conductance levels and heart rate variability across the waiting, phone and interaction phase.** Heart rate variability is represented in normalized units. Outliers are denoted by black dots and defined as points further than 1.5 × interquartile range of the lower or upper hinge.

**Fig. 6:**
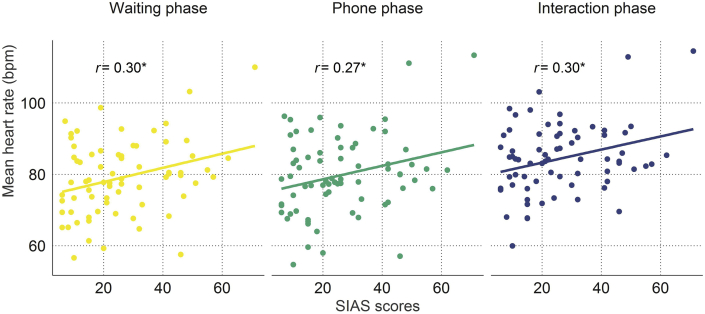
**Mean heart rate in beats per minute as a function of social interaction anxiety scale (SIAS) scores per phase.** Asterisks denote *p* < .05.

To investigate whether mean HR differed across phases and was impacted by social anxiety, we calculated an ANCOVA with the three-level factor experimental phase (waiting, phone and interaction phase) and the continuous SIAS score as covariate. A main effect of phase confirmed that heart rate differed between experimental phases (*F*_(2,138)_ = 60.48, ε = 0.78, *p* < .001, η^2^ = 0.05, see Fig. 5 and Supplemental Table S2) and a main effect of SIAS revealed that social anxiety was also associated with heart rate levels (*F*_(1,69)_ = 6.57, *p =* .01, η^2^ = 0.08). The interaction term did not reach statistical significance (*F*_(2,138)_ = 0.09, ε = 0.78, *p =* .92, η^2^ = 0.000), indicating that there was a stable influence of social anxiety on heart rate independent of the phase. To further assess the relationship between heart rate and social anxiety, we calculated Pearson's correlation coefficients comparing the association between SIAS scores and mean heart rate for each phase individually. Indeed, SIAS scores were significantly correlated with mean heart rate across all phases (waiting phase: *r* = 0.30, *p* = .012; phone phase: *r* = 0.27, *p* = .024; interaction phase: *r* = 0.30, *p* = .012, see Fig. 6).

An analogous ANCOVA model investigating influences of social anxiety and experimental phase on HF-HRV revealed that only the main effect of experimental phase was statistically significant (*F*_(2,136)_ = 20.24, ε = 0.96, *p* < .001, η^2^ = 0.10, see Fig. 5), while the main effect of SIAS (*F*_(1,68)_ = 0.03, *p =* .87, η^2^ < 0.001) and the interaction term (*F*_(2,136)_ = 0.19, ε = 0.96, *p =* .83, η^2^ < 0.001) did not reach statistical significance (for post-hoc comparisons see Supplemental Table S3).

Similar results were obtained regarding the influences of social anxiety and experimental phase on mean SCL. Skin conductance levels varied across phase as revealed by a main effect of experimental phase (*F*_(2,138)_ = 106.34, ε = 0.72, *p* < .001, η^2^ = 0.15, see Fig. 5 and Supplemental Table S4) but we neither observed a significant effect of SIAS (*F*_(1,69)_ = 0.11, *p =* .75, η^2^ = 0.001), nor of the interaction term (*F*_(2,138)_ = 0.95, ε = 0.72, *p =* .39, η^2^ = 0.002). For this reason, we did not conduct any follow-up correlational analyses between social anxiety and HF-HRV or SCL, respectively.

## 4 Discussion

Most of our current knowledge on the behavioral and physiological manifestation of social anxiety is based on the processing of simple social stimuli in a laboratory setting. The aim of the present study was to investigate autonomic responses and gaze behavior in individuals with differing degrees of (sub-)clinical social anxiety in a naturalistic scenario while manipulating the degree to which a social interaction is likely to develop. Participants were not informed about the start of the recordings but waited to start a different experiment while another participant, the confederate, filled in questionnaires. We generally found that, independent of social anxiety, head fixations were most prominently reduced when a social interaction was possible but likely undesired, as in this initial waiting phase. By subsequently rendering the confederate unavailable to talk through responding to a phone call, fixations on his head were seen to significantly increase. The likelihood of the confederate looking back or initiating a conversation hence seems to strongly affect gaze behavior. Overall, the final interaction, in which the confederate openly sought a conversation with the participant, was most demanding, as indicated by elevated heart rate and skin conductance levels and a reduced heart rate variability. Surprisingly, social anxiety levels did not impact gaze behavior in any of the phases of the experiment. With regard to physiological measurements, however, heart rate consistently correlated with social anxiety scores independent of the phase. A possible explanation is that individuals with (sub-)clinical social anxiety might be able to behaviorally compensate their fear of direct eye contact at the expense of increased physiological arousal in the presence of other people. These findings represent an important addition to observations made in simplistic laboratory scenarios.

By immersing the participants into a naturalistic setting without their awareness of experiment and recording commencement, we were able to gain essential insights into the development of gaze contact throughout a real social interaction. The presence of another person had previously been shown to induce norm-following and facilitate differential task processing (Guerin, 1986; Zajonc, 1965). As hypothesized by Laidlaw and colleagues (Laidlaw et al., 2011) who also investigated eye movements during a waiting room situation, our results confirm that the chance of interacting with another unknown person influences gaze behavior. We observed increased fixations on the head of the confederate when the possibility of an interaction decreased (i.e. when the confederate was occupied with a phone call). While cardiovascular responses did not differ between the waiting and the phone phase, heart rate was significantly more elevated and heart rate variability significantly decreased during the conversation with the confederate. Skin conductance levels already increased slightly when the confederate started talking on the phone, possibly because of heightened attention to the sudden auditory stimulation. Electrodermal activity was also highest during the interaction, emphasizing that conversations with strangers seem to come at cost of certain physiological arousal. It is arguably easiest to adhere to social norms by avoiding conversations in a waiting room scenario with strangers through reduced eye contact (Lalljee, 1978).

Considering the large body of conflicting literature on gaze behavior in social anxiety, the current results aid the interpretation of findings from simulated social exchanges and their potential translation to live social interactions. In line with studies drawing on gaze recordings during the viewing of photographed or video-animated faces (Beidel, Turner, & Dancu, 1985; Boll, Bartholomaeus, Peter, Lupke, & Gamer, 2016; Glasgow & Arkowitz, 1975; Hofmann, Gerlach, Wender, & Roth, 1997; Walters & Hope, 1998; Weeks, Heimberg, & Heuer, 2011), we did not observe any evidence for gaze avoidance in social anxiety. That is, we did not find reduced fixations on the head or body of the confederate in any of the experimental phases for individuals with higher levels of social anxiety. How can this finding be reconciled with the reported observations of gaze avoidance in social anxiety (Farabee et al., 1993; Howell et al., 2016; Langer et al., 2017; Moukheiber et al., 2010)? Social settings are diverse and different social encounters might facilitate distinct behavioral reactions. Possibly, the presence of a real person induces norm-activating behavior (Guerin, 1986; Zajonc, 1965), which, specifically during conversations, requires a certain level of attention towards the interaction partner. The use of images or videos as a simulation of an interaction might fail to enforce this heightened attention on the interaction partner in socially anxious individuals. Furthermore, the degree to which the social situation is perceived as threatening varies widely across reported experiments. We deliberately chose to investigate a neutral everyday situation to be able to draw inferences about non-threatening daily encounters. Previously, observed reductions in gaze in live interactions were often in response to conflicts or a disagreeing confederate (Farabee et al., 1993; Langer et al., 2017).

The elevated heart rate we observed in socially anxious participants could leave one to wonder whether overcoming fear of eye contact might ensue at the cost of physiological arousal. As this association has also been observed in another study with regard to electrodermal activity (Myllyneva et al., 2015), it is conceivable that social anxiety is primarily associated with either compensatory or concomitant physiological mechanisms in real interactions. It is unknown why we exclusively observed significant correlations between heart rate and social anxiety and not with any of the other physiological measures. The literature on physiological responses in social anxiety has been inconsistent and it is plausible that this inconsistency and our failure to observe altered electrodermal activity and heart rate variability in social anxiety stems from too low statistical power and the investigation of subclinical samples.

While the use of an everyday interaction allows us to investigate which alleged features of social anxiety survive outside of a standard laboratory setting, several limitations need to be considered. We aimed at maximizing the naturalness of our experimental design, which is why we decided against very standardized conversations between confederate and participant, a specific sequence of direct gaze or gaze avoidance of the confederate or a forced conflict situation. Consequently, we are unable to draw inferences about specific reactions to evaluative gaze or interactions with different emotional valence. Additionally, it is important to bear in mind that the confederate's head was also the sole source of auditory information during the phone and interaction phase. The auditory stimulation rather than the change in interaction possibility could have impacted the participant's increased attention. Future studies should therefore consider including a non-social auditory source to allow comparisons in attentional capture. While physiological reactions were most prominently increased in the conversation phase suggesting that this part of the experiment was also the most arousing to participants, an increase of movement during speech could also contribute to these observations. As social anxiety was yet associated with increased heart rate throughout the entire experiment, the association between physiological responses and anxiety symptoms cannot be fully explained by movement artefacts.

This finding nevertheless needs to be taken with a grain of salt since we are unable to conclude whether the increased heart rate is caused by the impending or current social interaction or by a higher baseline heart rate in socially anxious participants. The participation in an experiment inevitably induces brief social contact with the experimenter, even if no fellow participant is in the room, and it is therefore very difficult to isolate the causes of the observed heart rate increase. Future research should nevertheless aim to elucidate the circumstances under which elevated heart rates can be observed in socially anxious participants. Such examination might include methods of ambulatory assessment to examine heart rate changes across a variety of everyday situations in socially anxious participants (Sperry, Kwapil, Eddington, & Silvia, 2018). Additionally, socially anxious participants in our study were not clinically diagnosed and altered gaze patterns might only become apparent in a clinical sample. However, 26 out of the 71 participants demonstrated high SIAS scores (30 or higher) that also occur in clinical samples (Stangier et al., 1999). Since we observed virtually no correlations between social anxiety symptoms and measures of gaze (see Supplementary Fig. S3), the current study does not provide evidence for the assumption that results would look different in a clinical sample. Whether our failure to observe gaze differences in social anxiety reflects a true and stable effect of similar viewing behavior in anxious and non-anxious populations remains to be elucidated by future studies. Interestingly, non-clinical socially anxious participants responded with increased gaze to social cues when social evaluation was enhanced (Müller-Pinzler et al., 2015), suggesting that differences in gaze between socially anxious and non-anxious groups might only become apparent when the perceived social evaluation is sufficiently high. Controlling for state anxiety and the level of perceived social threat might help to interpret gaze patterns in the future.

The current results present evidence for increased heart rate but not for differences in gaze behavior in social anxiety during a real social interaction. Considering the inconsistent literature on social attention in socially anxious individuals, the current null findings suggest that gaze reduction might be either restricted to specific situations (e.g., including evaluative threat) or very high (clinically relevant) levels of social anxiety. The reported fear of eye contact might therefore at times remain a subjective feature of the disorder instead of translating into an observable behavioral measure. By contrast, increases in cardiovascular responses in social situations seem to represent a cardinal feature of social anxiety and current and future therapeutic interventions could thus consider targeting the awareness and regulation of cardiovascular activity.

## Funding

This work was supported by the 10.13039/501100000781European Research Council (ERC-2013-StG-336305) and the German Research Foundation (Collaborative Research Center “Fear, Anxiety, Anxiety Disorders”, SFB-TRR 58 project C10).

## CRediT authorship contribution statement

**Lara Rösler:** Conceptualization, Methodology, Data curation, Writing - original draft, Visualization, Investigation, Writing - review & editing. **Stefan Göhring:** Conceptualization, Methodology, Data curation, Writing - review & editing. **Michael Strunz:** Conceptualization, Methodology, Data curation, Writing - review & editing. **Matthias Gamer:** Conceptualization, Methodology, Supervision, Writing - review & editing.

## Declaration of competing interest

None.
