# Supplementary material for: Social anxiety is associated with heart rate but not gaze behavior in a real social interaction
Source: J Behav Ther Exp Psychiatry. 2021 Mar;70:101600. doi: 10.1016/j.jbtep.2020.101600 (PMC7689581; doi:10.1016/j.jbtep.2020.101600)
Supplement: Multimedia component 1 [file mmc1.pdf]

Social anxiety is associated with heart rate but not gaze behavior in a real social interaction

Lara Rösler, Stefan Göhring, Michael Strunz, Matthias Gamer

Department of Psychology, Julius Maximilians University of Würzburg, Würzburg, Germany

Supplementary Material

Fixation proportions on eye and mouth region

A large part of the literature on gaze behavior in social anxiety does not focus on head but on eye fixations (e.g., Horley et al. 2004; Boll et al. 2016; Moukheiber et al. 2012; Horley et al. 2003; Moukheiber et al. 2010). To investigate whether we can observe a relationship between social anxiety and gaze behavior with a more fine-grained spatial resolution, we re-coded fixations such that head fixations were labeled as either eye or mouth fixations. The nose tip was used to determine higher or lower face regions which were respectively labeled as eye or mouth region. The reported results yet need to be interpreted with caution because we cannot confidently state that the eye-tracking procedure employed in our study allows for such high resolution. While the accuracy of the glasses in itself should be high enough to distinguish between upper and lower face regions at a distance of approximately 2.5 meters, a three-point validation at the end of the experiment revealed gaze drifts in virtually all participants. On average, even after exclusion of the most extreme drift outliers, the absolute value of the gaze drift amounted to 10 (SD = 12) pixels on the x- and 37 pixels (SD = 39) on the y-axis. We applied a linear drift correction on our data assuming that the drift increases linearly throughout the experiment. As this is an assumption which we cannot test, fixations of the eye and the mouth region might be hard to tell apart (see Figure S1).

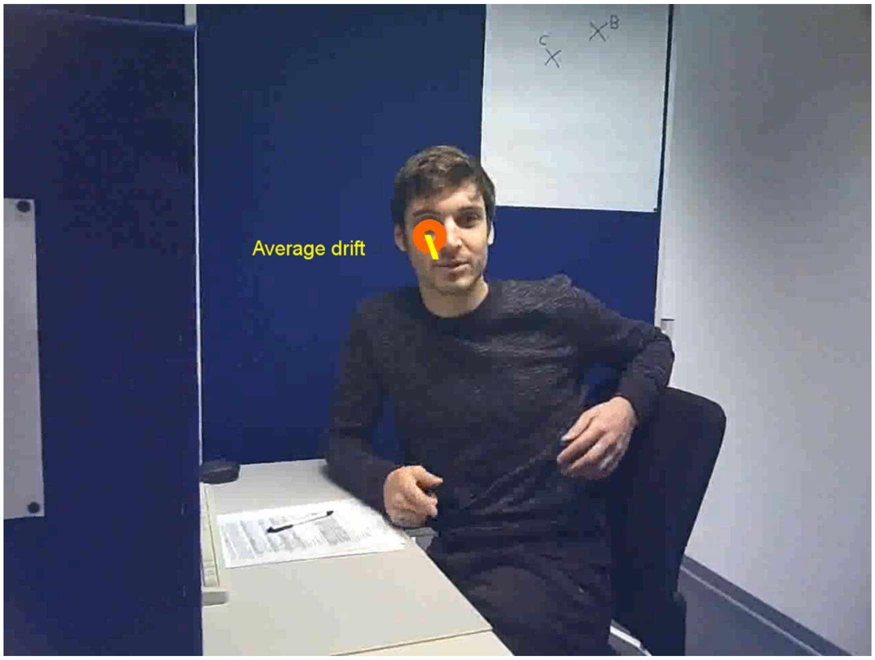

Figure S1. View of the confederate from the position of the participant. The yellow line indicates the average drift gaze across participants (x = 10 pixels, y = 37 pixels, here depicted as a negative value because the large majority of drifts on the y-axis were negative).

We nevertheless performed an ANCOVA with fixation proportions as the dependent variable, ROI (eyes versus mouth) and experimental phase (waiting, phone and interaction phase) as factorial predictors and SIAS score and gender as covariates. We found a significant main effect of ROI ($F_{(1,68)} = 6.56$, $p = .013$, $\eta^2 = .10$), as fixation proportions differed overall between mouth and eye regions (see Figure S2). Higher fixations on eye and mouth regions in the interaction phase become apparent in a significant main effect of experiment phase ($F_{(2,136)} = 165.15$, $\varepsilon = 0.79$, $p < .001$, $\eta^2 = .37$). We also observed a significant interaction of experiment phase and ROI ($F_{(2,136)} = 3.92$, $\varepsilon = 0.58$, $p = .045$, $\eta^2 = .42$) which mainly describes a larger increase of mouth as compared to eye fixations throughout the three experiment phases (see Figure S2). Social anxiety did not seem to have a significant effect on fixation proportions ($F_{(1,68)} = 0.62$, $p = .433$, $\eta^2 = .004$) and none of the individual interactions with experimental phase and ROI, nor the triple interaction between all three predictors or the effects of gender reached statistical significance (all $p > .32$).

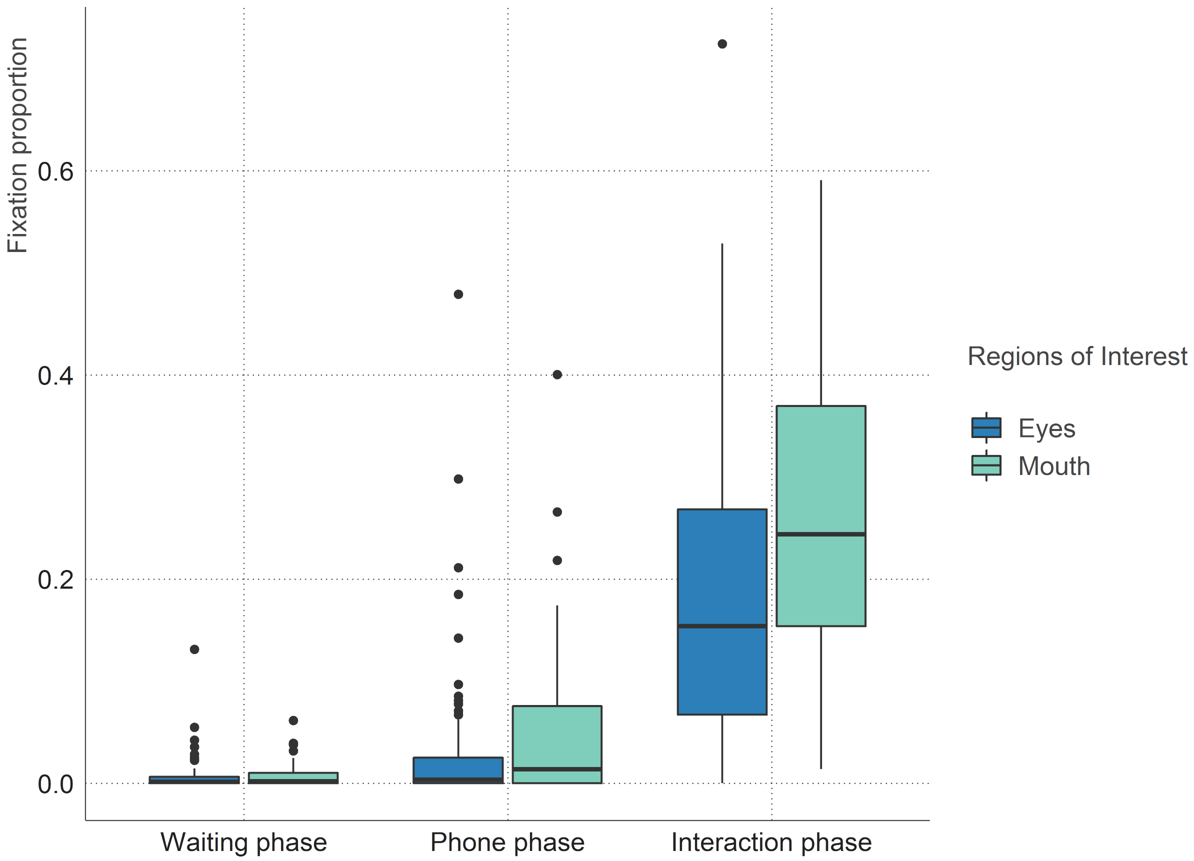

Figure S2. Eye and mouth region fixation proportions during waiting, phone and interaction phase. Outliers are denoted by black dots and defined as points further than 1.5 * interquartile range of the lower or upper hinge

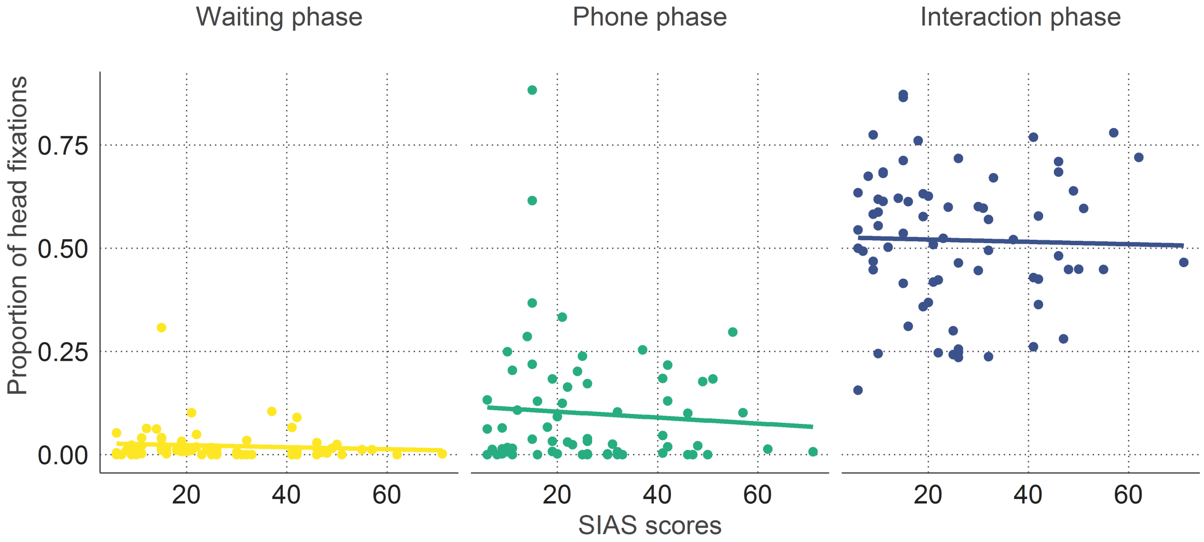

Figure S3. Mean fixation proportion on heads as a function of social interaction anxiety scale (SIAS) scores per phase.

Possible effects of gender on fixation proportion and physiological responses

As we preselected our sample according to social anxiety scores and higher levels are more prevalent in females, gender is gravely unbalanced in our sample (10 male, 61 female participants) rendering analyses of potential effects of gender on our dependent variables difficult. Additionally, the perceived gender of both of our confederates is male, so we cannot test any influences of the gender of the interaction partner. We decided, however, to re-run our analyses including gender as a covariate to correct for potential biases in our analyses induced by gender effects. Overall, we only observed a significant effect of gender on heart rate variability and none of the reported results changed in direction or significance level. Results as reported in the main body of the manuscript but now including gender as a covariate can be found below.

Gaze data

To investigate how fixation proportions on the confederate are impacted by social anxiety, experiment phase and ROI we performed an ANCOVA with fixation proportions as the dependent variable and ROI (head versus body) and experimental phase (waiting, phone and interaction phase) as factorial predictors and SIAS score and gender as covariates. We found a significant main effect of ROI ($F_{(1,68)} = 19.01$, $p < .001$, $\eta^2 = .07$) as there were overall considerable differences between body and head fixations (see Figure 3 and Supplemental Table S4). A significant main effect of experiment phase ($F_{(2,136)} = 101.54$, $\varepsilon = 0.95$, $p < .001$, $\eta^2 = .21$) is driven by higher fixation densities on the confederate during the interaction phase (see Supplemental Table S4). Importantly, we observed a significant interaction of experiment phase and ROI ($F_{(2,136)} = 101.94$, $\varepsilon = 0.88$, $p < .001$, $\eta^2 = .30$) which mainly describes an increase of head fixations throughout the experiment (see Figure 3 and Supplemental Table S4). Regarding potential influences of social anxiety on fixation proportion, we did not observe a significant main effect ($F_{(1,68)} = 1.03$, $p =.313$, $\eta^2 = .004$) and none of the individual interactions with experimental phase and ROI, nor the triple interaction between all three predictors reached statistical significance (all $p > .49$).

Physiological data

To investigate whether mean HR differed across phases and was impacted by social anxiety, we calculated an ANCOVA with the three-level factor experimental phase (waiting, phone and interaction phase) and the continuous SIAS score and the factorial variable gender as covariates. A main effect of phase confirmed that heart rate differed between experimental phases ($F(2,136) = 22.12$, $\varepsilon = 0.78$, $p < .001$, $\eta2 = .02$, see Figure 4 and Supplemental Table S5) and a main effect of SIAS revealed that social anxiety was also associated with heart rate levels ($F(1,68) = 6.54$, $p = .01$, $\eta2 = .08$). The interaction term did not reach

statistical significance ($F(2,136) = 0.08$, $\varepsilon = 0.78$, $p = .87$, $\eta 2 < .001$), indicating that there was a stable influence of social anxiety on heart rate independent of the phase. The main effect of gender ($F(1,68) = 0.45$, $p = .51$, $\eta 2 = .006$) and the interaction term with experimental phase ($F(2,136) = 1.22$, $\varepsilon = 0.78$, $p = .29$, $\eta 2 = .001$) did not reach statistical significance. To further assess the relationship between heart rate and social anxiety, we calculated Pearson's correlation coefficients comparing the association between SIAS scores and mean heart rate for each phase individually. Indeed, SIAS scores were significantly correlated with mean heart rate across all phases (waiting phase: $r = 0.30$, $p = .012$; phone phase: $r = 0.27$, $p = .024$; interaction phase: $r = 0.30$, $p = .012$, see Figure 5).

An analogous ANCOVA model investigating influences of social anxiety and experimental phase on HF-HRV revealed that only the main effect of experimental phase was statistically significant ($F(2,134) = 7.38$, $\varepsilon = 0.96$, $p = .001$, $\eta 2 = .04$), while the main effect of SIAS ($F(1,67) = 0.03$, $p = .86$, $\eta 2 < .001$) and the interaction term ($F(2,134) = 0.19$, $\varepsilon = 0.96$, $p = .82$, $\eta 2 = .001$) did not reach statistical significance (for post-hoc comparisons see Supplemental Table S6). Moreover, there was a significant main effect of gender on HF-HRV ($F(1,67) = 4.80$, $p = .03$, $\eta 2 = .04$) but not of its interaction with experimental phase ($F(2,134) = 0.38$, $\varepsilon = 0.96$, $p = .67$, $\eta 2 = .002$).

Similar results were obtained regarding the influences of social anxiety and experimental phase on mean SCL. Skin conductance levels varied across phase as revealed by a main effect of experimental phase ($F(2,136) = 37.76$, $\varepsilon = 0.72$, $p < .001$, $\eta 2 = .060$) but we did neither observe a significant effect of SIAS ($F(1,68) = 0.10$, $p = .75$, $\eta 2 = .001$), nor for the interaction term ($F(2,136) = 0.96$, $\varepsilon = 0.72$, $p = .36$, $\eta 2 = .001$). There was no statistically significant effect of gender ($F(1,68) = 0.45$, $p = .51$, $\eta 2 = .006$), nor of its interaction with experimental phase ($F(2,136) = 3.41$, $\varepsilon = 0.72$, $p = .05$, $\eta 2 = .005$) on SCL (for post-hoc comparisons see Supplemental Table S7). For this reason, we did not conduct any follow-up correlational analyses between social anxiety and HF-HRV or SCL, respectively.

Table S1. *Post-hoc contrasts of the interaction effect between ROI and experimental phase on fixation proportion, all p-values are Tukey-corrected for multiple comparisons.*

Contrast	*Estimate*	*SE*	*df*	*t*	*p*
Waiting.phase,head - Phone.phase,head	-0.107	0.028	257.660	-3.888	0.002
Waiting.phase,head - Interaction.phase,head	-0.513	0.028	257.660	-18.591	0.000
Waiting.phase,head - Waiting.phase,body	-0.178	0.035	187.133	-5.133	0.000
Waiting.phase,head - Phone.phase,body	-0.054	0.032	186.115	-1.699	0.534
Waiting.phase,head - Interaction.phase,body	-0.077	0.032	186.115	-2.423	0.154
Phone.phase,head - Interaction.phase,head	-0.406	0.028	257.660	-14.702	0.000
Phone.phase,head - Waiting.phase,body	-0.070	0.032	186.115	-2.201	0.242
Phone.phase,head - Phone.phase,body	0.053	0.035	187.133	1.538	0.640
Phone.phase,head - Interaction.phase,body	0.030	0.032	186.115	0.945	0.934
Interaction.phase,head - Waiting.phase,body	0.336	0.032	186.115	10.532	0.000
Interaction.phase,head - Phone.phase,body	0.459	0.032	186.115	14.402	0.000
Interaction.phase,head - Interaction.phase,body	0.436	0.035	187.133	12.608	0.000
Waiting.phase,body - Phone.phase,body	0.123	0.028	257.660	4.468	0.000
Waiting.phase,body - Interaction.phase,body	0.100	0.028	257.660	3.632	0.005
Phone.phase,body - Interaction.phase,body	-0.023	0.028	257.660	-0.836	0.961

Table S2. *Post-hoc contrasts for experimental phase on heart rate, all p-values are Tukey-corrected for multiple comparisons.*

Contrast	*Estimate*	*SE*	*df*	*t*	*p*
Waiting.phase - Phone.phase	-0.256	0.755	136.000	-0.339	0.939
Waiting.phase - Interaction.phase	-4.472	0.755	136.000	-5.922	0.000
Phone.phase - Interaction.phase	-4.217	0.755	136.000	-5.584	0.000

Table S3. *Post-hoc contrasts for experimental phase on heart rate variability, all p-values are Tukey-corrected for multiple comparisons.*

Contrast	*Estimate*	*SE*	*df*	*t*	*p*
Waiting.phase - Phone.phase	-1.292	2.939	134.000	-0.440	0.899
Waiting.phase - Interaction.phase	9.069	2.939	134.000	3.085	0.007
Phone.phase - Interaction.phase	10.361	2.939	134.000	3.525	0.002

Table S4. *Post-hoc contrasts for experimental phase on skin conductance level, all p-values are Tukey-corrected for multiple comparisons.*

Contrast	*Estimate*	*SE*	*df*	*t*	*p*
Waiting.phase - Phone.phase	-0.908	0.384	136.000	-2.362	0.051
Waiting.phase - Interaction.phase	-3.238	0.384	136.000	-8.423	0.000
Phone.phase - Interaction.phase	-2.330	0.384	136.000	-6.061	0.000
